# Signal amplification in molecular sensing by imprinted polymers

**DOI:** 10.1007/s00604-024-06649-x

**Published:** 2024-09-04

**Authors:** Mingli Chen, Haiyan Li, Xiaoting Xue, Fang Tan, Lei Ye

**Affiliations:** 1 Research Center for Analytical Sciences, Department of Chemistry, College of Sciences, Northeastern University, BOX 332, Shenyang, Liaoning 110819 P.R. China; 2 Division of Pure and Applied Biochemistry, Department of Chemistry, Lund University, Box124, 22100 Lund, Sweden; 3 School of Optoelectronic Materials & Technology, Jianghan University, Wuhan, Hubei 430056 P.R. China

**Keywords:** Molecular imprinting polymers (MIPs), Electrochemical/optical sensors, Signal amplification, Biomolecules

## Abstract

In the field of sensing, the development of sensors with high sensitivity, accuracy, selectivity, sustainability, simplicity, and low cost remains a key focus. Over the past decades, optical and electrochemical sensors based on molecular imprinting techniques have garnered significant attention due to the above advantages. Molecular imprinting technology utilizes molecularly imprinted polymers (MIPs) to mimic the specific recognition capabilities of enzymes or antibodies for target molecules. Recently, MIP-based sensors rooting in signal amplification techniques have been employed to enhance molecular detection level and the quantitative ability for environmental pollutants, biomolecules, therapeutic compounds, bacteria, and viruses. The signal amplification techniques involved in MIP-based sensors mainly cover nucleic acid chain amplification, enzyme-catalyzed cascade, introduction of high-performance nanomaterials, and rapid chemical reactions. The amplified analytical signals are centered around electrochemical, fluorescence, colorimetric, and surface-enhanced Raman techniques, which can effectively realize the determination of some low-abundance targets in biological samples. This review highlights the recent advancements of electrochemical/optical sensors based on molecular imprinting integrated with various signal amplification strategies and their dedication to the study of trace biomolecules. Finally, future research directions on developing multidimensional output signals of MIP-based sensors and introducing multiple signal amplification strategies are proposed.

**Graphical abstract:**

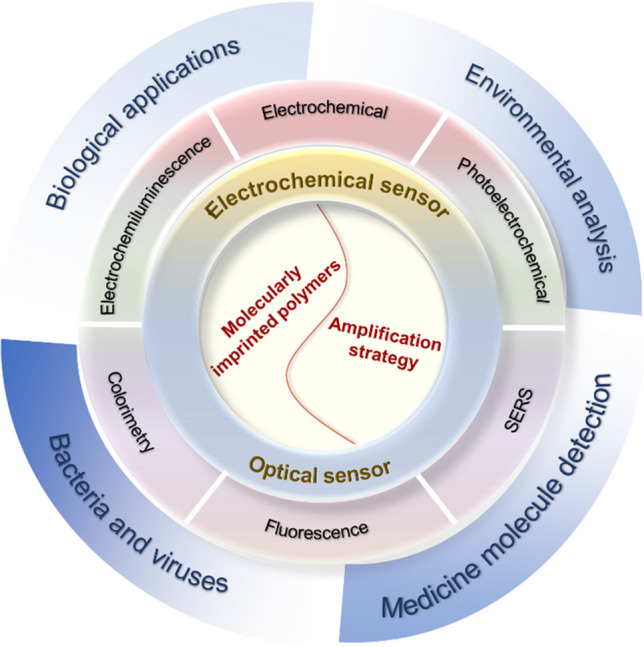

## Introduction

Molecular imprinting technology (MIT) provides a practical approach for constructing highly selective adsorbent materials. Its mechanism effectively mimics the natural antigen–antibody interaction, often described as a “lock and key” configuration. The molecular imprinting process involves polymerizing functional monomers and cross-linking them in the presence of a molecular template. Upon removal of the template, the resulting cavities are complementary to the template in terms of shape, size, and chemical functionality. As a result, molecularly imprinted polymers (MIPs) possess pre-defined recognition sites capable of binding target molecules with high selectivity [1]. The main methods for synthesizing molecularly imprinted polymers (MIPs) include bulk polymerization, precipitation polymerization, microemulsion polymerization, suspension polymerization, multi-step swelling polymerization, and in situ electropolymerization. During these polymerization processes, imprinted cavities are formed within crosslinked polymers, which can be used for the specific recognition of molecular targets. Over the past decades, MIPs have been applied in various fields, including as sorbents for chromatographic separation, affinity matrices for solid-phase extraction, molecular sensing, cell imaging, and therapy, among others [2–5]. As a unique type of polymer with tailor-designed specificity, molecularly imprinted polymers have become an indispensable material for constructing chemical sensors due to their excellent molecular recognition capabilities, stability, and cost-effective production.

Unlike biological receptors, MIPs are better suited for use under harsh environmental conditions that can cause irreversible denaturation of proteins and nucleic acids. As a result, MIPs are often selected as molecular recognition elements in the development of biomimetic sensors, which play a crucial role in analytical sciences related to environmental, clinical, and food applications. MIPs with target-specific recognition have demonstrated exceptional performance in various electrochemical and optical sensors due to the wide applicability of the output signal. Among these, MIP-based optical sensors utilize different spectral signals, such as fluorescence, UV–vis, and surface-enhanced Raman scattering, for detection and quantification. Electrochemical sensors based on MIPs are characterized by high selectivity, high sensitivity, low cost, and ease of miniaturization and automation. On the other hand, MIP-based optical sensors offer high stability and good repeatability. A variety of signal transduction schemes have been employed in MIP-based sensors for applications such as environmental monitoring, biomarker analysis, and food safety control [6, 7].

Given the complexity of biological systems and the growing need for lower detection limits in life science and environmental research, it has become crucial to monitor low-abundance substances in complex samples. This necessitates highly sensitive and specific MIP-based sensors. To overcome these challenges, signal amplification strategies have been integrated with molecularly imprinted polymers, enabling effective identification and quantification of analytical targets. Signal amplification is primarily achieved by constructing functional interfaces and designing specialized molecular probes to enhance sensitivity and achieve low detection limits. To translate the limited number of molecular binding events into detectable signals, various functional nanomaterials, nucleic acids, enzyme cascade, and specific chemical reactions are incorporated into the analytical systems, which are activated by the specific molecular binding [8]. With the rapid advancement of nanotechnology, nanomaterials with high specific surface area, excellent biocompatibility, and outstanding electrical conductivity have been utilized for signal amplification in biosensors by providing abundant reactive sites or accelerating reaction centers for molecular imprinting. These nanomaterials are categorized based on their structures into zero-dimensional, one-dimensional, two-dimensional, and three-dimensional materials, represented by nanoparticles, nanowires, nanosheets, and porous nanoflowers, among others [9]. Current nucleic acid amplification strategies include polymerase chain reaction (PCR), rolling circle amplification (RCA), hybridization chain reaction (HCR), ligase chain reaction (LCR), and loop-mediated isothermal amplification (LAMP). Nucleic acid amplification in MIPs refers to the replication or hybridization of DNA fragments on the polymer through enzymatic or non-enzymatic reactions to produce amplified output signals [10]. Enzyme cascade involves a system where an intermediate product triggers multiple subsequent catalytic reactions, leading to amplified signals. This often includes a sequence of enzyme-catalyzed reactions that form a reaction pathway, enabling significant signal enhancement [11]. In addition, rapid chemical reactions, such as click reactions, have become popular methods for signal amplification due to their speed and high specificity. Integrating MIPs with appropriate signal amplification techniques can significantly enhance the capability of biomimetic sensors to detect low-abundance analytes in complex systems.

**Scheme 1:**
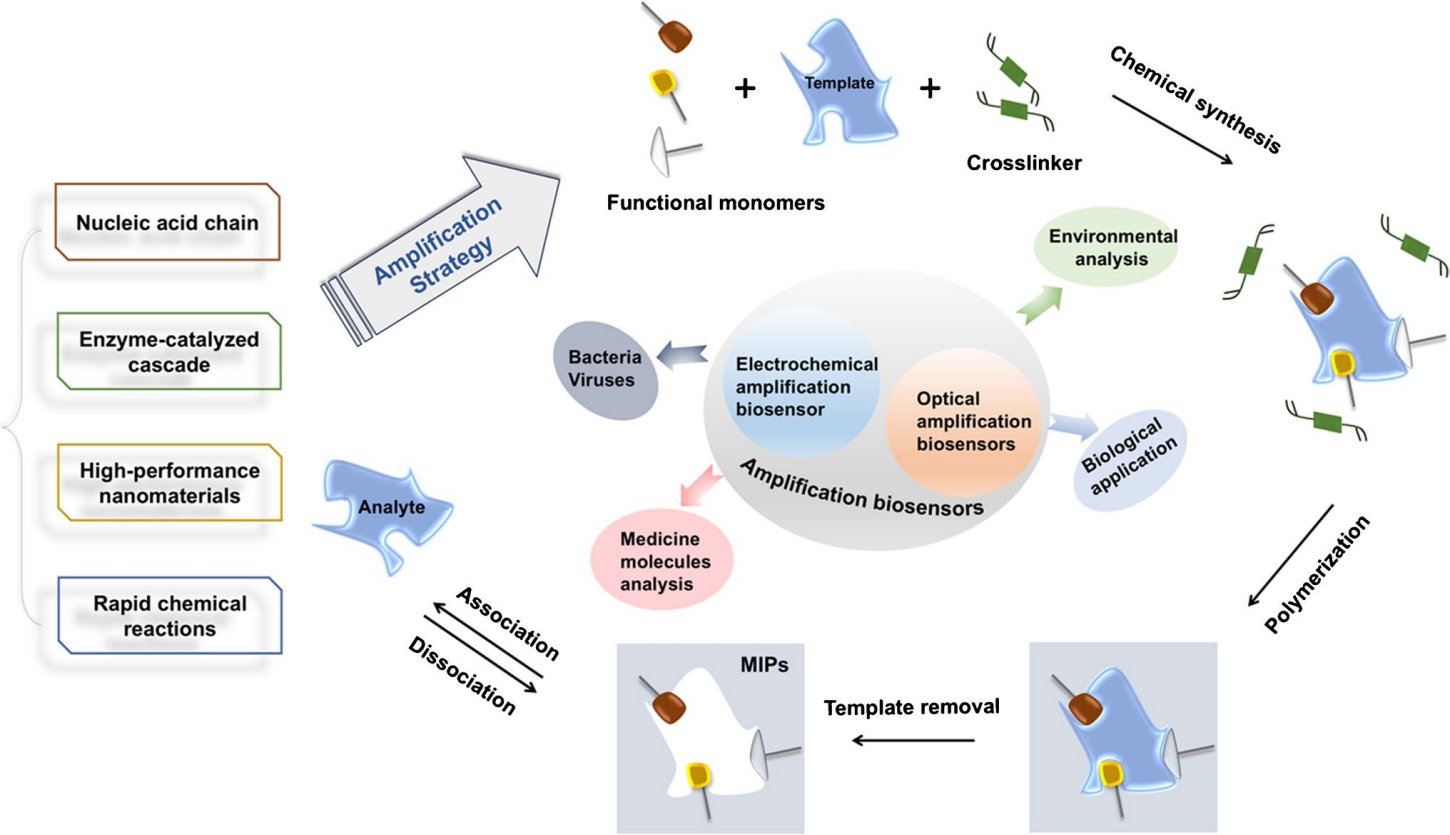
Preparation scheme of molecularly imprinted polymers and the application of MIP-based amplification biosensors

This mini-review will focus on the past 5 years of research on MIP-based sensors that incorporate various signal amplification techniques, as described in Scheme 1. The analytical systems discussed will primarily revolve around MIP-based electrochemical and optical sensors designed for a broad range of applications such as environmental monitoring, diagnostics, and the detection of pathogenic microorganisms. Representative examples will be highlighted, followed by an analysis of current challenges and potential future developments in the field.

## Electrochemical signal amplification for biochemical detection

Signal-amplified electrochemical (EC) biosensors based on molecularly imprinted polymers (MIPs) hold significant practical value in biochemical sensing. The primary approach to enhancing analytical sensitivity in these sensors is the incorporation of nanomaterials with high electrical conductivity or excellent chemical activity as signal amplifiers. In this section, we will review recent advances in MIP-based electrochemical sensors that utilize signal amplification strategies. Table 1 provides an overview of the various methods employed for the preparation of MIP-based electrochemical sensors with signal amplification capabilities.

**Table 1:** The recent advances of MIP-based electrochemical and optical sensors with signal amplification

MIP-based senor	Techniques	Substrate	Linearity range	LOD	Ref
Fe_3_O_4_@Pt NPs/COF-AIECL@MIP	Electrochemical	Ciprofloxacin	2 × 10^−12^–3 × 10^−9^ M	5.98 × 10^−13^ M	[12]
MIP-PEC sensor	Electrochemical	Acrylamide	0.25–10000 nmol	1.7 pmol	[13]
PDA@Au NCs-MIPs	Electrochemical	Formaldehyde	0.2 μM–0.02 M	0.1 μM	[14]
MIPs/Te–CdS@Mn_3_O_4_/GCE,	Electrochemical	2,4-D	1 nM–100 μM	0.63 nM	[15]
MIP/AAO	Electrochemical	Myocardial troponin T	1.08 × 10^–4^–4.25 × 10^–4^ ng/mL	5.34 pg/mL	[16]
GCE/MIP@SAA/SiO2@Au@Ab	Electrochemical	Ovalbumin	1.0 × 10^−11^–1.0 × 10^−7^ mg/mL	3.0 fg/mL	[17]
MIP@CoZn-NC/GCE	Electrochemical	Uric acid	1–300 μM	0.41 μΜ	[18]
MIPs/Au-Pt NMs/SPCE	Electrochemical	C-reactive protein	0.1 nM–500 nM	0.1 nM	[19]
MIP/CuCo_2_O_4_/NCNTs/FC/GCE	Electrochemical	Gemcitabine	0.1–150 μM	11.3 nM	[20]
MIP/pTHi/Ni-MOF/Fe-MOF-5/AuNPs/GCE	Electrochemical	Chlorpromazine	0.001–40 μM and 40–900 μM	0.025 μΜ	[21]
SPCE-MWCNT-AgNP-MIP	Electrochemical	MDPV	–	1.8 μM	[22]
PPy/CuPcTs/MIPs	Electrochemical	*Escherichia coli*	10^2^–10^7^ CFU/mL	21 CFU/mL	[23]
Ti_2_C doping pEIPs-coated	Electrochemical	SARS-CoV-2	0.01–1000 fg/mL	%1.%2 fg/mL	[24]
Electrodes					
Mg,N-CDs/Eu-MOFs@MIP	Fluorescence	Oxytetracycline	0.02–50 μg/mL	6.6 ng/mL	[30]
MOF-Apt@MIP	Fluorescence	Malachite green	0.1–10 ng/mL	0.1 ng/mL	[31]
Melanin@N-GQDs@MIP	Fluorescence	Prometryn	1.0–100.0 μg/kg	0.63 μg/kg	[32]
*m*-CD@SiO_2_/MIP	Fluorescence	Pyrethroids	1–150 μg/L	0.048 μg/L	[33]
Mg, N-CDs/r-CdTe@UiO-66@MIP	Fluorescence	2,4,6-Trinitrophenol	1–100 μM	0.56 μΜ	[35]
FIS@MIP	Fluorescence	Transferrin	0.1–20 μM	0.067 μM	[36]
MIP/MSN/HCR	Fluorescence	Hemoglobin	0.01–1 mg/mL	0.006 mg/mL	[37]
MIP/Fe_3_O_4_/EGP	Colorimetric	Aloe-emodin	5.0 × 10^−8^–1.0 × 10^−4^M	3.8 × 10^−8^ M	[39]
THPP@MIP	Colorimetric	Etoposide	0.005–10 μg/mL	0.002 μg/mL	[40]
MIP/HKUST-1	Colorimetric	TBBPA	0.01–10 ng/g	3 pg/g	[41]
U6NH@AuNPs-ChOx@MIPs	Colorimetric	Cholesterol	2.9–6.7 mM	2.4 mM	[42]
HAuCl_4_-DA-MIP	SERS	Acetamiprid	0.25–20 pM	–	[43]
MOFTb@Au@MIP	SERS	Malathion	–	0.06 ng/mL	[44]
AuNR@MIP	SERS	Rhodamine B	2 × 10^−8^–10^−6^ M	2 × 10^−8^ M	[45]
SiO2@AuAg@rMIP@CAP/MOFs@Au@TB@Apt	SERS	CAP	1.0 × 10^−12^–1.0 × 10^−6^ M	7.59 × 10^−13^ M	[46]
MMIPs NPs	SPR	Tetracycline	5.0–100 pg/mL	1.0 pg/mL	[47]
MAA/SSS-MMIPs NPs	SPR	3-MPA	5–300 pg/mL	3.02 pg/mL	[48]

### Environmental sample analysis

**Fig. 1:**
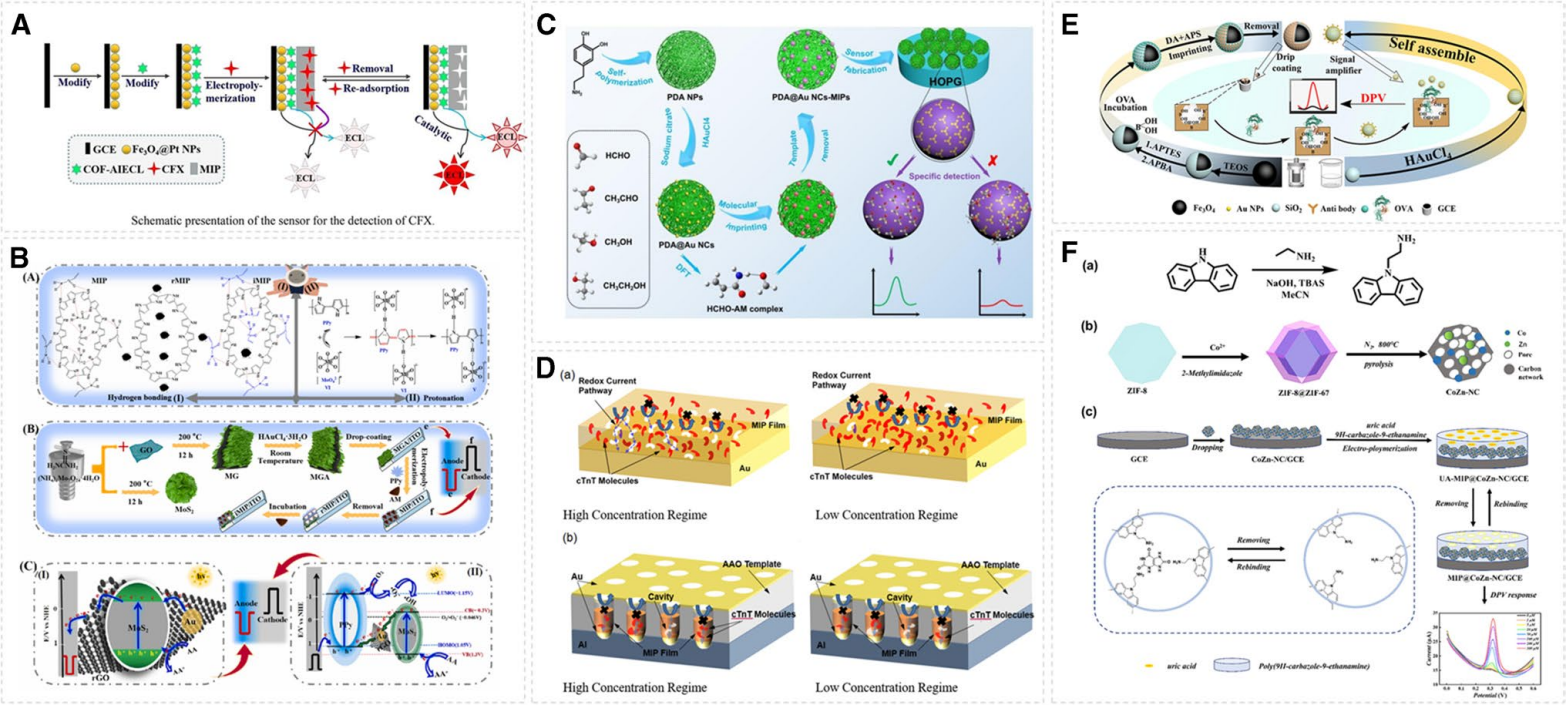
Construction of molecularly imprinted electrochemical amplification sensor for detecting environmental pollutants and biological molecules. **A** MIP-based sensor for the detection of CFX. Reprinted with permission from ref. [12]. Copyright 2022 Elsevier. **B** The construction of polarity-switchable MIP-PEC sensor with “Z-scheme.” Reprinted with permission from ref. [13]. Copyright 2024 Elsevier. **C** The construction of PDA@Au NCs-MIPs for formaldehyde detection. Reprinted with permission from ref. [14]. Copyright 2021 Elsevier. **D** The construction of MIP-based cardiac troponin T (cTnT)-sensing electrode. Reprinted with permission from ref. [16]. Copyright 2021 American Chemical Society. **E** The preparation process of the MIP-based electrochemical sensor for OVA detection. Reprinted with permission from ref. [17]. Copyright 2023 Elsevier. **F** The schematic representation of the MIP@CoZn-NC/GCE sensor for uric acid measurement. Reprinted with permission from ref. [18]. Copyright 2024 Elsevier

Electrochemical signal amplification sensors based on molecularly imprinted polymers have demonstrated significant advantages in the detection of environmental pollutants. For example, as depicted in Fig. 1A, a novel molecularly imprinted sensor was developed using a covalent organic framework (COF) combined with aggregation-induced electrochemiluminescence (AIECL) for the sensitive detection of ciprofloxacin (CFX) [12]. The highly conductive Fe₃O₄@Pt nanoparticles were employed as electrochemiluminescence (ECL) signal amplifiers and were initially used to modify the electrode surface. Subsequently, molecularly imprinted polymers were synthesized on the modified electrode using ciprofloxacin (CFX) as the template molecule. The aggregation-induced electrochemiluminescence (AIECL) sensor was then utilized to determine picomolar level CFX residues in veterinary drugs. As illustrated in Fig. 1B, a polarity-switchable photoelectrochemical sensor was developed based on a “Z-scheme” strategy for the detection of acrylamide (AM) [13]. The MoS_2_/rGO/Au (MGA) with a large specific surface area and good electrical conductivity was adopted as the substrate and polypyrrole (PPy) as the functional monomer. AM was used as the template molecule to construct the MIP-coated electrodes. Herein, PPy also acted as a polarity switcher to establish a Z scheme in the MIP-PEC system and generated a cathode photocurrent. For sensitive determination of formaldehyde down to micromolar level, a signal amplification system based on the integration of dual high conductivity gold nanoclusters (AuNCs) and polydopamine nanospheres (PDA NPs) was developed (Fig. 1C), where acrylamide was adopted as the functional monomer [14]. Also, a ratiometric electrochemiluminescence sensing platform was constructed for detecting 2,4-dichlorophenoxyacetic acid (2,4-D) at nanomolar level, where Te–CdS@Mn_3_O_4_ nanozyme accompanied with excellent electrochemical effect was used to improve the ECL signal.

### Biological molecule analysis

Electrochemical sensor-coupled MIPs enable highly selective determination of specific biomolecules, including proteins, neurotransmitters, uric acid, etc. These biological molecules are important for the operation of physiological functions. An anodic molecular-imprinted nanocomposite electrode was fabricated using a process compatible with semiconductors, and high-conductivity alumina (AAO) was added to effectively improve the electrode performance [16], and the sensitivity of cardiac troponin T was measured at the level of nanograms per milliliters (Fig. 1D). Utilizing anti-ovalbumin antibody modified gold nanoparticles as amplifiers, an ingenious sandwich-structured electrochemical sensor was designed for ovalbumin (OVA) detection at the level of femtogram per milliliters [17]. Imprinted cavities on magnetic particles capture target proteins efficiently using boric acid affinity, prompting the gold nanoparticle amplifier to form a sandwich structure. This sandwich assay showed superior selectivity and sensitivity to OVA, as shown in Fig. 1E. A procedure was designed for uric acid measurement employing a molecularly imprinted polymer and porous Co/Zn embedded, N-doped carbon material (Fig. 1F) [18]. By electropolymerization of carbazole derivatives, a polymer with a large number of amino groups was obtained to increase the number of binding sites for uric acid, which enhanced the selectivity of the sensor. The porous CoZn-NC polyhedral nanocages provide a highly conductive sensing interface that promotes the catalytic oxidation of uric acid and achieves a lower detection limit. Utilizing MIP fabricated on an electrode surface, an electrochemical sensor for detecting C-reactive protein was described [19]. The screen-printed carbon electrode coated with gold-platinum bimetallic nanomaterials improved the surface area and catalytic performance of the sensor, while the MIP coating on the sensing platform facilitates its selectivity and sensitivity.

### Therapeutic drug assays

**Fig. 2:**
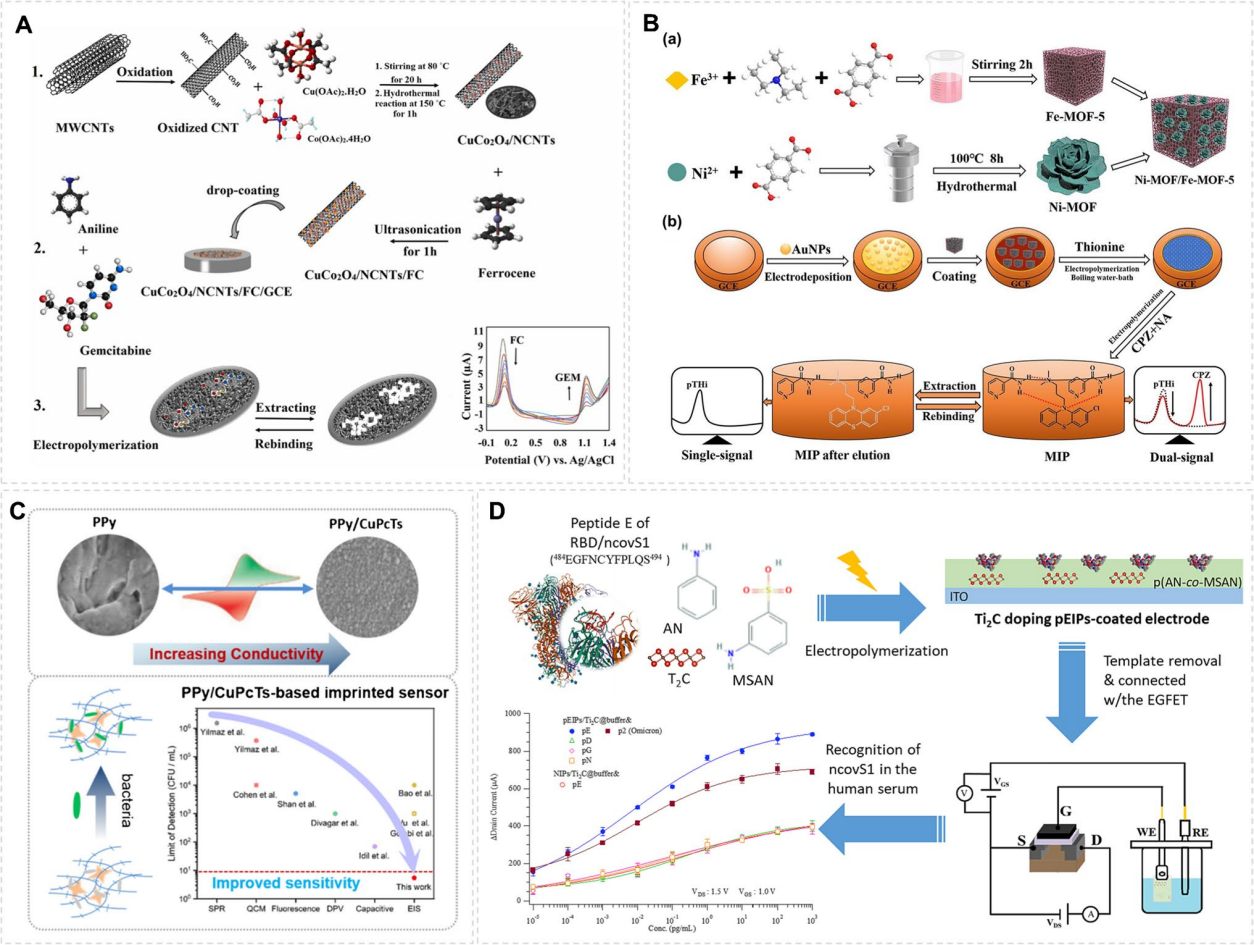
Construction of molecularly imprinted electrochemical amplification sensor for detecting therapeutic molecules, bacteria, and viruses. **A** The fabrication process of the MIP/CuCo_2_O_4_/NCNTs/FC/GCE sensor for electrochemical detection of GE. Reprinted with permission from ref. [20]. Copyright 2022 Elsevier. **B** Preparation procedure of MIP/pTHi/Ni-MOF/Fe-MOF-5/AuNPs on–off electrochemical sensor. Reprinted with permission from ref. [21]. Copyright 2023 Elsevier. **C** The construction of a novel bacteria-imprinted polymer sensor for the measurement of Escherichia coli. Reprinted with permission from ref [23]. Copyright 2023 Springer. **D** Preparation of SARS-CoV-2 receptor-binding domain (RBD) peptide-imprinted poly(AN-co-MSAN) coated electrodes. Reprinted with permission from ref. [24]. Copyright 2023 Elsevier

The detection method based on MIP has shown specific selectivity and utility in the analysis of some therapeutic drugs. An electrochemical method was developed for the determination of gemcitabine, an anticancer drug [20]. Gemcitabine-imprinted polymer was prepared by in situ electrochemical polymerization of aniline. A glassy carbon electrode was modified with CuCo_2_O_4_/NCNTs and ferrocene to achieve a ratiometric on–off response, where CuCo_2_O_4_/NCNTs/FC served as a signal amplifier based on high transition metal-induced conductivity (Fig. 2A). For detection of the antipsychotic medication chlorpromazine, nicotinamide was used as a functional monomer and in situ polymerized in the presence of chlorpromazine [21]. AuNPs, Ni-MOF/Fe-MOF-5, and polythionine were used as internal reference to achieve the ratiometric on–off response, in which the composite materials contributed to amplifying the output signal based on its own porous conductive matrix (Fig. 2B). A sensor for 3,4-methylenedioxypyrovalerone, a dopamine transporter blocker [22] was constructed by combing electropolymerized MIP with AgNPs and multi-walled carbon nanotubes. Benzene-1,2-diamine served as the functional monomer and the target analyte as the template monomer. The MWCNT-AgNP-MIP sensor is rooted in high conductivity to achieve effective signal amplification and to detect 3,4-methylene dioxy pentanone at micromolar concentrations.

### Bacteria and viruses

The detection of bacteria and viruses is of great significance for the prevention of disease transmission, and sensors based on molecular imprinting have outstanding potential in the area. *Escherichia coli* was measured by a bacteria-imprinted impedimetric sensor [23]. The labelless sensor was prepared by one-step electropolymerization of pyrrole and cupric phthalocyanine 3,4′,4″,4″-tetrasodium tetrasulfonate (CuPcTs) on glassy carbon electrodes in the presence of target bacteria. The PPy/CuPcTs surface contains imprinted sites that bind specifically to the target bacteria and produce impedance changes upon bacterial binding. On the sensing surface, the PPy/CuPcTs structure resulted in excellent conductivity for improving the sensitivity of the sensor. To detect the virus, an epitope-imprinted conductive polymer was integrated with an extended-gate field-effect transistor to develop a novel sensor for the detection of COVID spike proteins [24]. The imprinted polymer (AN-co-MSAN) was synthesized by electropolymerization using the peptide epitope on the spike protein as a template. The electrochemical signal generated by the analytical target was enhanced by doping highly conductive titanium carbide (Ti_2_C) into the conductive polymer film. Electrochemical amplification sensors based on MIPs have also exhibited excellent ability to detect trace level of toxic metal ions [25, 26].

## Optical signal amplification for biochemical sensing

Optical sensors are widely applied in various fields including environmental analysis, biological application, analysis and measurement of therapeutic molecules, bacteria, and viruses. Fluorescence, colorimetric, and Raman signals are the most common optical sensor readings. Optical signals have high sensitivity, stability, and flexibility, which combined with the specificity of MIPs can effectively sense different analysis targets [27–29]. There are three different approaches to building an MIP-based optical sensor. One is to use functional monomers with observable optical properties to synthesize the MIPs; the second one is to coat MIPs on the surface of an optical material; the third one is to synthesize composite materials containing MIPs and luminescent nanoparticles. This section summarizes recent advances in optical sensors based on MIPs combined with signal amplification strategies. Table 1 provides an overview of the various fabrication methods of MIP-based optical amplification sensors and their applications.

### Fluorescent MIP sensors

**Fig. 3:**
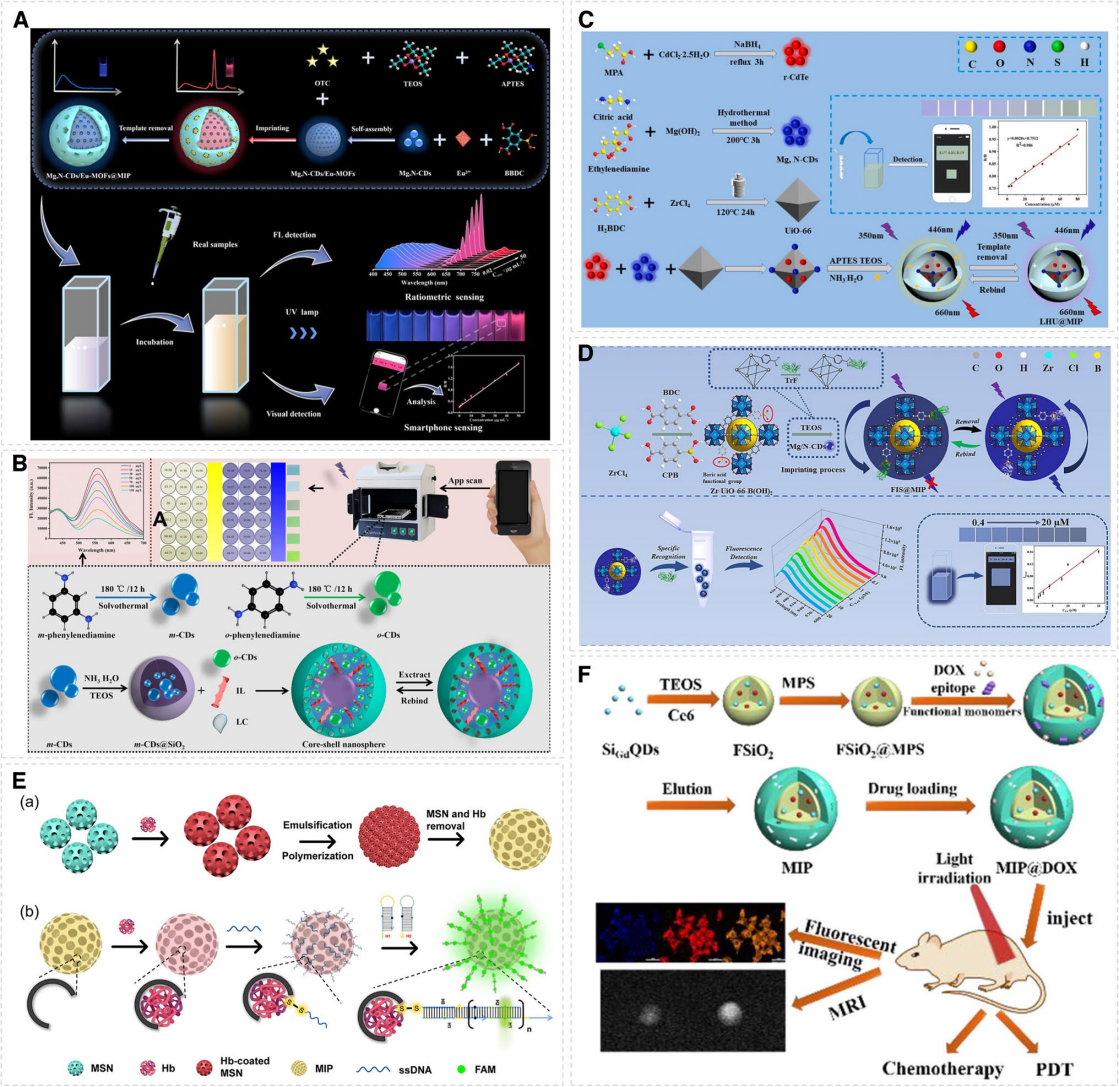
Construction of molecularly imprinted fluorescence sensors. **A** The preparation process of Mg, N-CDs/Eu-MOFs@MIP with ratiometric fluorescence for detecting oxytetracycline. Reprinted with permission from ref. [30]. Copyright 2022 Elsevier. **D** The preparation of core–shell nanospheres for ratiometric sensing λ-Cyhalothrin. Reprinted with permission from ref [33]. Copyright 2022 Elsevier. **C** The synthetic process of LHU@MIP and smartphone-assisted detection of TNP. Reprinted with permission from ref [35]. Copyright 2024 Elsevier. **D** The preparation process of FIS@MIP for the detection of TrF. Reprinted with permission from ref. [36]. Copyright 2024 Elsevier. **E** Combination strategy of MIP and HCR for high-sensitivity detection of Hb. Reprinted with permission from ref [37]. Copyright Open Access. **F** The preparation of MIPs@DOX for targeted chemo-photodynamic synergistic treatment of tumor in vivo. Reprinted with permission from ref. [38]. Copyright 2023 American Chemical Society

Fluorescent MIP sensors combine the high sensitivity of fluorescence and the selectivity of MIPs and are attractive for the development of sensitive methods for various analytical targets. Commonly used fluorescent materials or probes include carbon dots, quantum dots, and other fluorophores, which were combined with MIPs to construct sensors. Based on the synergic effect of inner filter effect (IFE) and antenna effect (AE) [30], a terramycin field monitoring proportional fluorescence sensor was constructed [30]. The high surface area europium metal–organic skeleton (Mg, N-CDs/Eu-MOFs) can be embedded with rich optical carbon dot labels, and then, the sol–gel technology can be used to coat the surface of the fluorescent particles with MIP layer for specific and highly sensitive detection (Fig. 3A). A paper-based fluorescence sensor for malachite green was designed based on a metal–organic framework capped with aptamer and MIP, where the efficient binding of aptamer and its specificity highlighted the signals to improve the detection sensitivity [31]. The green fluorescent terbium metal–organic framework served as a carrier to immobilize a malachite green specific aptamer, which was subsequently coated with a MIP layer imprinted against malachite green. A fluorescent nanoprobe for prometryn measurement was constructed using melanin nanoparticles as an affinity material, which could improve the adsorption of prometryn on the nanoprobe [32]. Nitrogen-doped graphene quantum dots (N-GQDs) were used to improve the response sensitivity and load the MIP layer to achieve selectivity. For determination of λ-cyhalothrin, a dual-channel ratiometric fluorescence strategy was adopted using blue-green dual-emission carbon dots. λ-Cyhalothrin was used as the template to synthesize the MIP layer. Ionic liquids with a wide viscosity range and good thermal stability were used in the analytical system to improve the detection sensitivity (Fig. 3B).

A smartphone-integrated tri-color fluorescence sensor was constructed based on acid-sensitive fluorescence-imprinted polymers fabricated by sol–gel polymerization for visual detection of ibuprofen, chloramphenicol and florfenicol [34]. Fluorescence and RGB values were recorded via a smartphone with high selectivity and sensitivity for the three target analytes. A ratiometric fluorescence sensor for 2,4,6-trinitrophenol measurement was constructed by combining imprinted polymers with magnesium-nitrogen co-doped carbon dots and chromium telluride quantum dots [35]. The use of zirconium-based organic scaffolds (UiO-66) with large surface area and porosity provides abundant imprinting sites, while Mg, N-CDs, and r-CdTe with high quantum yields are selected as fluorophores to facilitate signal amplification (Fig. 3C). For measurement of transferrin (TrF), a fluorescent MIP sensor [36] was demonstrated in which Mg/N co-doped carbon dots with high fluorescence quantum yield was used as fluorophore and boronic acid functionalized Zr-MOF as signal booster to improve the specificity and sensitivity (Fig. 3D). For detection of hemoglobin, surface-imprinted polymer microspheres were combined with hybridization chain reaction (HCR) to achieve amplification of the fluorescence signal triggered by the protein binding [37]. The Hb-imprinted polymer beads were synthesized by Pickering emulsion polymerization, and mesoporous silica nanoparticle (MSN) was used as carriers to immobilize the protein template. For analytical detection, the captured Hb on the MIP surface was labeled with ssDNA that subsequently triggered HCR amplification of the fluorescence signal, as shown in Fig. 3E. A bifunctional imprinted polymer nanoparticle with a core–shell structure was prepared via free-radical precipitation polymerization on the surface of fluorescent silica nanoparticles (FSiO_2_) using an epitope imprinting approach [38]. The fluorescent nanoparticle core encapsulates gadolinium-doped silicon quantum dots and photosensitizer (Ce6). The embedded Ce6 produces toxic ^1^O_2_ upon photoexcitation, whereas the loaded therapeutic drug DOX has synergistic anticancer effects. Gadolinium-doped silicon quantum dots (QDs) enable targeted fluorescence imaging (FI) and magnetic resonance imaging (MRI).

### MIP-based colorimetric sensors

**Fig. 4:**
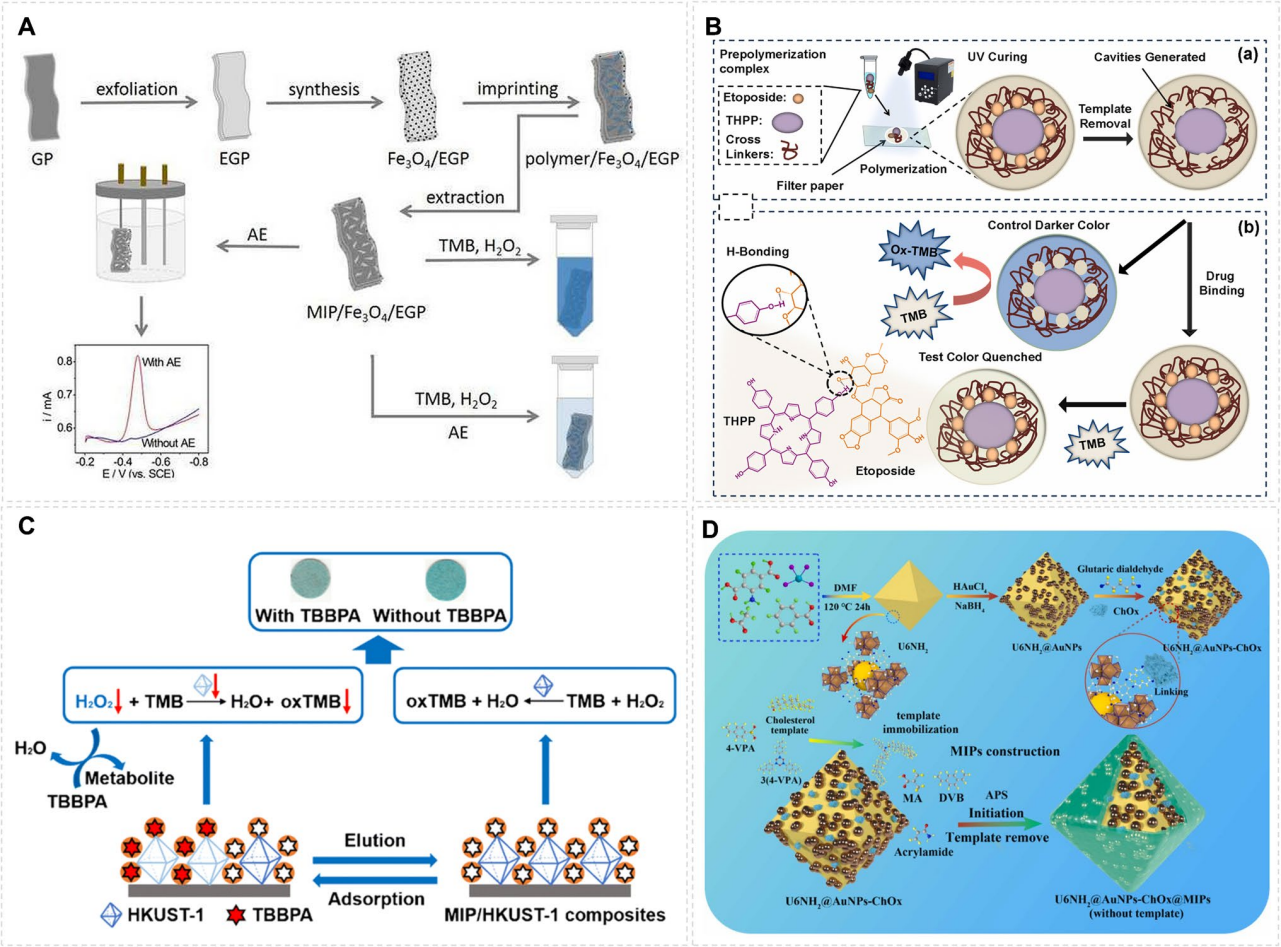
Construction of molecularly imprinted colorimetric amplification sensor for detecting biomolecules. **A** The fabrication process of MIP/Fe_3_O_4_/EGP with dual-modal strategy for the detection of AE. Reprinted with permission from ref [39]. Copyright 2020 Elsevier. **B** The representation of THPP@MIP sensor for the detection of ETO. Reprinted with permission from ref [40]. Copyright 2024 Elsevier. **C** The preparation of MIP/HKUST-1 composites and its application for TBBPA detection. Reprinted with permission from ref [41]. Copyright 2020 Springer. **D** The step-by-step construction of U6NH2@AuNPs-ChOx@MIPs for detecting cholesterol. Reprinted with permission from ref. [42]. Copyright 2024 Elsevier

The colorimetric method has the advantages of simplicity, low cost, fast analysis speed, etc., but it also faces the problem of large interference. Because of their molecular binding selectivity, MIPs have attracted much attention for developing colorimetric sensors and assays. The combination of colorimetric reaction with MIPs have led to a series of new sensors for biomolecular analysis. A novel electrochemical/colorimetric dual-modal sensor for detecting aloe-emodin (AE) was prepared [39] by combining in situ synthesized Fe_3_O_4_ with MIP on the surface of exfoliated graphite paper (EGP) (Fig. 4A). MIP/Fe_3_O_4_ microparticles exhibited peroxidase-like activity and selective recognition of the analytical target, which laid the foundation for colorimetric sensing. The multi-layer EGP with large surface area and excellent electrical conductivity promoted higher sensitivity of the system. For selective detection of etoposide (ETO) in lung cancer patients, an oxidase-like nanozyme was combined with MIP to achieve amplified colorimetric signal [40]. The colorimetric sensor (THPP@MIP) is fixed on the cellulose paper to simplify the analysis and inspection. THPP@MIP catalyzes the oxidation of colorless 3,3′,5,5′-tetramethylbenzidine (TMB), which yields a UV signal after binding to the target analyte (Fig. 4B). Using a copper-based metal–organic framework on a paper support, a colorimetric MIP sensor for tetrabromobisphenol A (TBBPA) was also demonstrated. TBBPA adsorbed on the MIP can degrade and consume H_2_O_2_ and decrease the catalytic activity of HKUST-1 underneath the MIP layer [41] (Fig. 4C). For detection of cholesterol, a colorimetric sensor was fabricated by using metal–organic skeleton (U6NH_2_) as a carrier to embed AuNPs and cholesterol oxidase (ChOx) [42]. A cholesterol-selective MIP was incorporated into the sensing material to enable the target-triggered cascade colorimetric reactions (Fig. 4D).

### MIP-based surface sensors

**Fig. 5:**
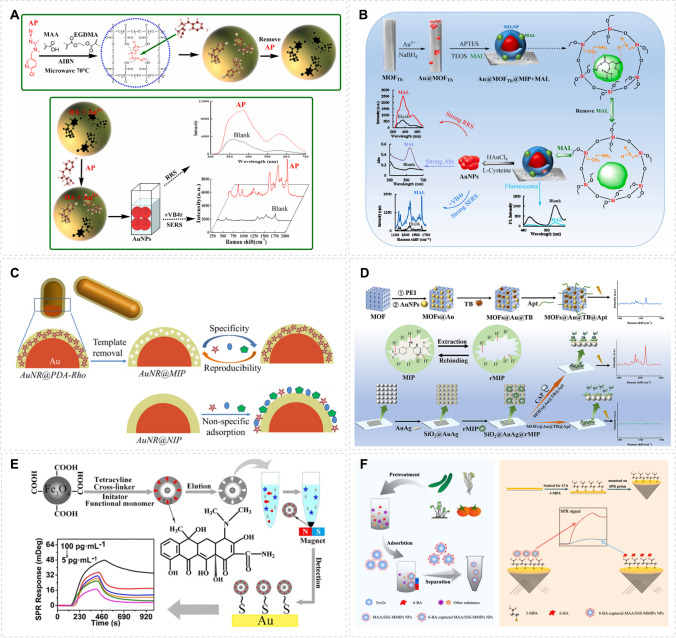
Construction of molecularly imprinted SERS amplification sensor for detecting biomolecules **A** The preparation of acetamiprid (AP) molecularly imprinted polymer (MIP) nanosol. Reprinted with permission from ref [43]. Copyright 2023 Elsevier. **B** The design of MOFTb@Au@MIP sensor for SERS determination of MAL. Reprinted with permission from ref [44]. Copyright 2024 Elsevier. **C** Presentation of the specific detection of rhodamine B based on AuNR@MIP sensor. Reprinted with permission from ref [45]. Copyright 2023 Springer. **D** The construction process of the SERS biosensor for detecting CAP. Reprinted with permission from ref [46]. Copyright 2024 Elsevier. (E) MMIPs NPs-based SPR sensor for the detection of tetracycline (TC). Reprinted with permission from ref. [47]. Copyright 2019 Springer. F The design of MIP-based SPR sensor for the detection of 6-benzylaminopurines. Reprinted with permission from ref [48]. Copyright 2024 Elsevier

Molecular imprinting combined with surface-responsive sensors, such as surface-enhanced Raman scattering (SERS) and surface plasmon resonance (SPR), have been widely used in biochemical analysis due to its ultra-high sensitivity, rapidness and nondestructive nature. The MIP-based surface response strategy has yielded a range of advanced biomolecular detection methods with promising applications. For detection of acetamiprid (AP), a bifunctional MIP-based sensor was prepared using a microwave reactor. The analyte in the picomolar range was detected by SERS and resonance Rayleigh scattering (RRS) [43]. Binding of AP to the bifunctional MIP made the material able to catalyze the nanogold dimode indicator reaction. The generated gold nanoparticles (AuNPs) acted as “hot spots” to produce surface-enhanced Raman scattering (SERS) signal (Fig. 5A). In another study, a bifunctional nanoprobe composed of metal–organic framework and MIP (MOFTb@Au@MIP) was developed for SPR detection of malathion [44]. The nanoprobe can specifically recognize malathion (MAL) and catalyze the l-cysteine (Cys)-HAuCl_4_ indicator to generate gold nanoparticles with SERS “hot spots” to amplify the spectral signal (Fig. 5B). A molecularly imprinted polymer/reduced graphene oxide (MIP/RGO)-based electrochemical sensor was constructed for determination of picomolar levels of vanillin (VAN). Using rhodamine B as a model, MIP-coated gold nanorod (AuNR@MIP) was constructed to act as a SERS sensor [45]. In this work, polydopamine (PDA) was used as the base material for imprinting with rhodamine B. The presence of AuNRs enhances the Raman output signal of the analytes for highly sensitive analysis (Fig. 5C). To realize ultrasensitive detection of chloramphenicol (CAP) with detection limit as low as sub-picomolar concentration, a SERS system was constructed by combining MIP with aptamer-functionalized SERS substrate (MOFs@Au@TB@Apt) [46]. The dual recognition of MIP and aptamer enables the sensitive determination of CAP (Fig. 5D). Magnetic molecular-imprinted polymer nanoparticles combined with surface plasmon resonance signals for high-sensitivity tetracycline sensors are proposed [47], which can significantly amplify signals depending on the high refractive index of the Au chip, as shown in Fig. 5E. Using methacrylic acid (MAA) and sodium p-styrene sulfonate (SSS) as imprinted functional monomers, the high-refractive index gold chip was used as the surface plasmon resonance (SPR) matrix to amplify the output signal [48], as shown in Fig. 5F. Magnetic molecularly imprinted polymer nanoparticles (MMIPs-NPs) coupled SPR sensors were developed for the highly sensitive detection of 6-benzylaminopurines (6-BA) in vegetables at concentrations as low as picograms per milliliters.

## Conclusions and prospects

Molecular imprinting has made significant advancements in the field of sensing, where molecularly imprinted polymers (MIPs) serve as antibody mimics to provide specific molecular recognition. The synthesized MIPs exhibit excellent resistance to high temperatures and pressures, as well as to acidic and alkaline environments, and they offer easy storage, making them highly promising for the analysis of complex samples. When combined with signal amplification functions, MIPs enable ultrasensitive detection of low-abundance substances in these complex matrices. The challenge of detection limits can be addressed by designing effective amplification pathways, such as the use of functional nanomaterials. Additionally, employing DNA amplification technology or cascade chemical reactions can greatly enhance the analytical signal induced by target binding. Amplification systems based on catalytic MIPs, such as MIP-functionalized nanozymes, are particularly promising and have already been demonstrated in various fields, including environmental analysis, therapeutic drug monitoring, and the detection of bacteria and viruses.

However, several challenges remain to be addressed in the coming years. For example, sensors designed with multiple detection channels for various targets may meet more practical demands, the multiple amplification strategies could be explored to enhance the amplification ratio to achieving more efficient determination, also integrating molecularly imprinted sensors with emerging technologies, such as artificial intelligence and the Internet of Things (IoT), could usher in a new era of intelligent, adaptive, and self-calibrating sensing systems.

## Data Availability

No datasets were generated or analysed during the current study.
